# Experiments with “Birron’s Antidote”

**Published:** 1858-10

**Authors:** 


*Experiments with "Bibron's Antidote."* By A. M. SABAL, M. D., Riceboro, Liberty County, Ga.

1*st Experiment.* This dog was bitten just at the sacro-lumbar articulation—was quite thin in flesh and weak. In four minutes from the reception of the wound, he staggered and fell—from that time he could no longer support himself—twelve minutes after he vomited blood freely. I now administered a dose of "Bibron's Antidote." He seemed to revive almost immediately and attempted to regain his feet, but his efforts proved abortive—fifteen minutes from the administration of the first dose, I exhibited a second (10 drops) of the mixture. He again revived for a few minutes but could not move. Twelve minutes afterwards six drops of the mixture was given him. He died in taking it.

This dog died in forty-three minutes from the time he was bitten; there was little or no swelling; his eyes became perfectly green several minutes before death; his tongue contracted to one-half its normal size and became of a dark, purplish color.

2*d Experiment.* This dog was fine, fat and hearty. He was caused to be bitten three times in the flank; did not show any symptoms of uneasiness for fifteen minutes, when all at once he began to swell rapidly, whine, and stretch himself, as if much distressed—ten drops of the Bromine mixture was administered and he seemed much better. Fifteen minutes after he began to be quite sick, froth at the mouth, &c.; a second dose was administered in same proportion as the first, he revived the moment he took it and has been well ever since with the exception of a swollen leg, which lasted twenty-four hours and gradually disappeared.

3*d Experiment.* This dog was caused to be bitten in the leg, in two different places, severely. He immediately became sick and vomited freely the contents of his stomach; subsequently he vomited frothy blood and bled at the nose. The antidote was administered twenty minutes from the reception of the wounds, in six drop doses and repeated every ten minutes until four doses were taken. This dog became perfectly well, but was quite sick for six hours, at the end of which time a fifth dose was given him. His leg remained swollen for two days.

4*th Experiment.* A dog, thin in flesh, but old, caused to be bitten in the side and foreleg. The medicine was administered as in all other cases—he lived thirty hours and died. This dog was enormously swollen after death; not so with any of the others that died.

*5th Experiment.* A young dog bitten in the neck. The dog lived five hours under the administration of the medicine, but being called off, I neglected him, and was informed he died apparently of suffocation.

*6th Experiment.* This dog was bitten in the flank, received all attention, and died in an hour and a-half.—*Savannah Journal of Medicine.*

